# Overexpressed CD24 and CD47 Indicate a Worse Prognosis in Cervical Cancer

**DOI:** 10.1002/cam4.71443

**Published:** 2025-12-07

**Authors:** Min Yu, Mengdong Ni, Fei Xu, Chi Fang, Jiajia Li, Xiaohua Wu, Guihao Ke

**Affiliations:** ^1^ Department of Gynecologic Oncology Fudan University Shanghai Cancer Center Shanghai China; ^2^ Department of Oncology Shanghai Medical College, Fudan University Shanghai China

**Keywords:** CD11c, CD24, CD47, cervical cancer, M1‐polarized macrophage

## Abstract

**Background:**

Two antiphagocytic (“don't eat me”) signals that allow tumor immune evasion have been discovered, including CD24 and CD47. This study explored the association between CD24/CD47 expression and macrophage infiltration and clinical outcomes in cervical cancer.

**Methods:**

RNA expression and survival data of the Cancer Genome Atlas (TCGA) cohort were extracted from OncoLnc. The macrophage infiltration level was calculated using xCell, TIMER, and ImmuCellAI. The expression of CD24 and CD47 was detected by immunohistochemistry in tissue microarrays composed of 130 clinical cervical cancer specimens from Fudan University Shanghai Cancer Center (FUSCC). Patients' medical records were also retrospectively assessed to correlate demographic and survival data.

**Results:**

Expression levels of both CD24 and CD47 in the cancer population were higher than those in the normal population. Patients with high CD24 expression had poorer survival than those with low CD24 expression in the TCGA and FUSCC cervical cancer cohorts. Although CD47 alone was not statistically significant in predicting outcomes, patients with high CD47 and low CD11c expression, a specific marker of M1‐polarized macrophages, exhibited worse survival in the TCGA cohort.

**Conclusions:**

Our study implies that high CD24 expression is an important predictor of a worse prognosis, and CD24 blockade might have therapeutic potential for the treatment of cervical cancer. High expression levels of CD47 and low M1‐polarized macrophage infiltration predict a worse prognosis.

## 1 Introduction

Cervical cancer (CC) is one of the leading causes of cancer‐related death in women worldwide [1]. Despite the significant development of HPV vaccines, there has been limited progress in the treatment of recurrent or advanced cervical cancer, with only 35%–50% response rates with current treatment options [2]. Cancer immunotherapy has emerged as one of the most promising therapeutic strategies, providing a novel option to fight cancer for cervical cancer patients [3, 4].

Many immune checkpoints have been identified and applied for clinical use, such as PD‐1/PD‐L1 and CTLA‐4/B7. These checkpoints regulate adaptive T cell‐mediated immunity against cancer [5, 6, 7]. Another type of innate immune checkpoint can enhance the activity of phagocytes, thereby promoting antigen‐presenting cells' function and stimulating the T cell‐mediated anti‐tumor immune response [8, 9]. This promising innate immune checkpoint is composed of CD47 and CD24 and their inhibitory immuno‐receptors signal regulatory protein α (SIRPα) and Siglec‐10, respectively [10, 11, 12]. CD47 is an immunoglobulin broadly overexpressed on the surface of cancer cells; CD47 inhibits macrophage‐mediated phagocytosis through binding to SIRPα on phagocytes. Many studies have confirmed that anti‐CD47 antibody can recover macrophage‐mediated phagocytosis in cancer and enhance an anti‐tumor T‐cell immune response [13, 14, 15]. Many clinical trials involve CD47‐targeting antibodies or recombinant proteins to block the CD47‐SIRPα signal in patients with advanced solid tumors or hematological cancers.

CD24, formerly known as glycosylated mucin‐like cell surface protein, is a newly identified antiphagocytic molecule that can be harnessed by cancer cells to avoid attack from Siglec‐10‐expressing macrophages [11]. CD24 was identified as a marker of cancer stem cells in various human malignancies and is involved in tumor metastasis and invasion [16, 17]. Its function in modulating tumor immune responses has recently been demonstrated in ovarian and breast cancers.

In this study, we integrated CD24 and CD47 into the analysis of cervical cancer from TCGA and Fudan University Shanghai Cancer Center (FUSCC). We explored the joint prognostic value of macrophages, which has rarely been reported in cervical cancer. Our results provide evidence for the therapeutic potential of CD24 and CD47 blockade, with particular promise for the treatment of cervical cancer.

## 2 Materials and Methods

### 2.1 Acquisition of Gene Expression and Survival Data of the TCGA Cohort

The overall survival (OS), living status, and corresponding expression of CD24/CD47 were retrieved from the OncoLnc [18]. OncoLnc (http://www.oncolnc.org/) contains survival data for 264 cervical cancer patients from TCGA and gene expression data from “rsem.genes.normalized_results” files.

### 2.2 Macrophage Infiltration Level Calculation

Tumor‐infiltrating immune cells in cervical cancer were quantified by Immune Cell Abundance Identifier (ImmuCellAI), a tool to estimate the abundance of immune cells based on gene expression datasets, including RNA‐Seq and microarray data [19]. The infiltration levels of macrophages and their subgroups, namely M1 and M2, were calculated by xCell. This gene signature‐based algorithm performs cell type enrichment analysis according to gene expression data [20].

### 2.3 Patients From FUSCC and the Data Collection

A total of 130 patients with cervical cancer who had undergone radical surgery were consecutively collected from FUSCC between 2014 and 2015. Stages were assigned based on the International Federation of Gynecology and Obstetrics (FIGO) surgical staging of cancer of the cervix uteri (2009). Patients with distal metastasis, additional malignancies, or a history of chemoradiotherapy were excluded. The final pathologic diagnosis was confirmed by two experienced gynecologic pathologists using World Health Organization (WHO) definitions. All 130 patients provided written informed consent for the use of tissue samples. The ethics committee at FUSCC also verified and approved the study.

### 2.4 Tissue Microarray (TMA) and Immunohistochemistry (IHC)

The TMAs containing duplicate 2.00‐mm cores from paraffin‐embedded tumor tissues were constructed at the FUSCC biorepository facility. Each tumor block was subjected to hematoxylin and eosin (H&E) staining. The immunostaining method used was avidin‐biotin immunoperoxidase. The tissue sections were deparaffinized in xylene (3 × 10 min) and rehydrated by graded alcohol dehydration (100%, 100%, 95%, and 75%). Heat‐mediated antigen retrieval was performed with citrate buffer (pH = 6.0) in a pressure cooker for 30 min. The endogenous peroxidase was exhausted with 100% methanol containing 0.3% H_2_O_2_ at room temperature for 25 min. After blocking in 5% bovine serum albumin (BSA) for 1 h, the sections were incubated with rabbit anti‐CD47 antibody (20305‐1‐AP, 1:200 dilution ratio; Proteintech, Rosemont, USA) and anti‐CD24 antibody (10600‐1‐AP, 1:200 dilution ratio; Proteintech) overnight at 4°C. Next, the sections were incubated with a biotinylated secondary antibody. The final staining was performed using avidin‐biotin‐peroxidase complex for 3 min, and then the samples were counterstained with hematoxylin.

The immunostaining results were examined by two experienced pathologists independently. CD24 and CD47‐positive samples showed membranous and cytoplasmic staining. The semi‐quantitative immunoreactivity scoring system (H‐score) was applied to evaluate the expression level of CD24 and CD47, which was derived from the multiplication of the percentage of positive tumor cells (P‐score, ranging from 0 to 100) and the intensity of staining (I‐score, ranging from 0 to 3; 0, no staining; 1, weak; 2, moderate, and 3, strong).

$$H-\text{score}=\Sigma \left(P-\text{score}\times I-\text{score}\right)$$

### 2.5 Statistical Analysis

Overall survival was defined as the duration from the surgery date to the date of death or the last follow‐up. Relapse‐free survival (RFS) was defined as the duration from the date of surgery to the date of disease relapse. Continuous variables such as CD24/CD47 expression level and macrophage infiltration level were dichotomized for OS before the Log‐rank test according to optimal cutoff values calculated by the “surv_cutpoint” function. The cutoff value was worked out using something called the “maximally selected rank statistic” from the survminerR package. The statistical association between clinicopathological features and CD24/CD47 expression levels was examined by the Pearson *χ* ^2^ test or Fisher's exact test. Kaplan–Meier method and Log‐rank test were employed in univariate analysis of RFS and OS, while a Cox proportional hazards model was adopted in the multivariate analysis. A two‐sided *p* value < 0.05 was considered statistically significant.

## 3 Results

### 3.1 CD24 and CD47 Expression in Cervical Cancers From TCGA

**FIGURE 1:**
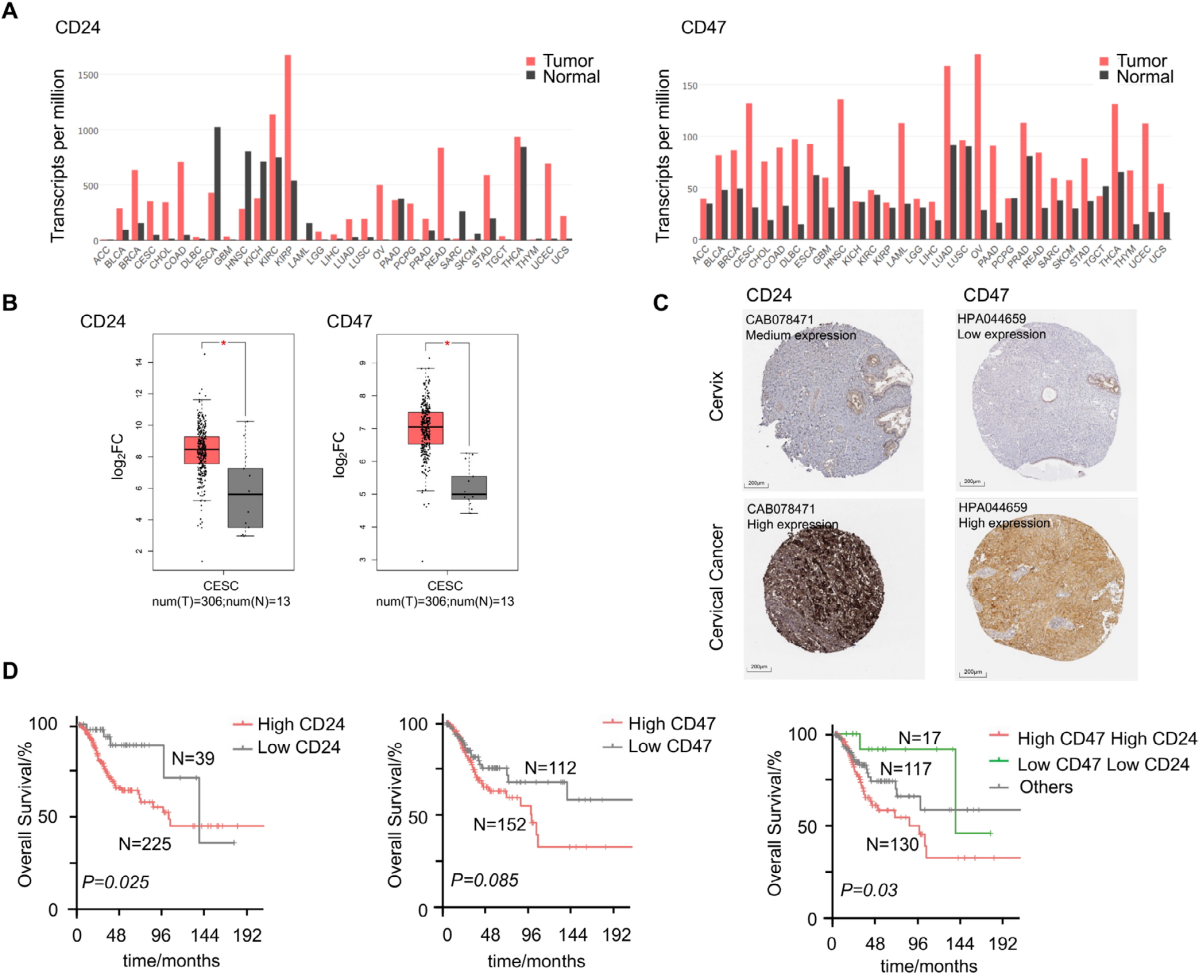
CD24 and CD47 expression in TCGA CC and corresponding association with OS. (A) Expression of CD24 and CD47 was extensively elevated across 31 cancer entities (red) compared to normal tissue (gray). (B) mRNA expression of both CD24 and CD47 was significantly higher in cancer samples than in normal samples (one‐way ANOVA, *p < 0.01). (C) Protein expression of CD24 and CD47 was higher in cancer samples than in normal samples (data from HPA). (D) High expression of CD24 was associated with poor OS (Log‐rank test, *p* = 0.025). Correlation between OS and expression level of CD47 wasn't significant (Log‐rank test, *p* = 0.085). Patients with overexpression of both CD24 and CD47 have the worst prognosis. Kaplan–Meier analysis of CC from TCGA (*n* = 264).

A pan‐cancer analysis of CD24/CD47 expression from TCGA showed an extensive overexpression across 31 tumor entities compared to normal tissues (Figure 1A). The mRNA expression of both CD24 and CD47 in TCGA‐CESC datasets exhibited statistical significance between cancerous and normal tissues. In contrast, statistical differences were not obtained across different tumor stages (Figure 1B and Figure S1A). Accordingly, the protein expression level of CD24/CD47 in cervical cancer was significantly higher than that of normal cervical tissue (Figure 1C). To investigate the prognostic potential of CD24 and CD47, we performed survival analysis for 264 cervical cancer cases whose survival data and sequencing results were obtained from OncoLnc. Consistent with previous studies in cervical cancer [21, 22], CD24 overexpression was preferentially associated with poor survival (*p* = 0.025, Figure 1D). As for CD47, overexpression of it showed a worse OS as indicated by survival curves, though the difference did not reach statistical significance (*p* = 0.085, Figure 1D). Correlation analysis showed that CD47 expression was positively associated with CD24 expression (*p* < 0.001, Figure S1B). High expression of CD24 and CD47 distinguished patients with better prognoses (*p* = 0.030, Figure 1D).

### 3.2 Macrophage Infiltration in Cervical Cancers From TCGA

**FIGURE 2:**
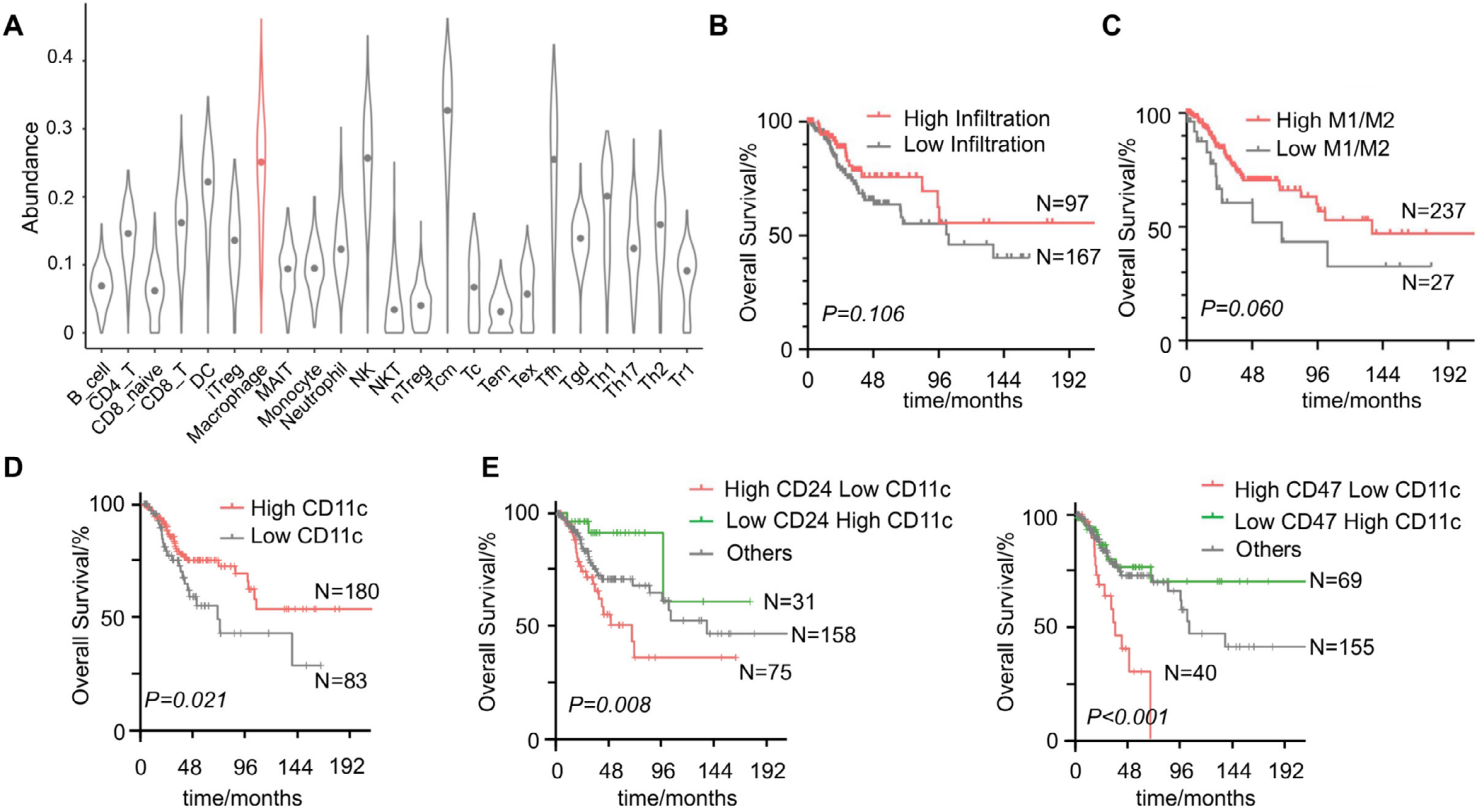
Prognostic value of M1‐polarized macrophage infiltration level and its correlation with CD24/CD47 expression. (A) The landscape of 23 tumor‐infiltrating immune cells in CESC was quantified by immuCellAI. (B) Patients with different infiltration levels of macrophages didn't show a significant difference in OS (Log‐rank test, *p* = 0.106). (C) Patients with a high M1/M2 infiltration ratio showed a tendency for worse OS (Log‐rank test, *p* = 0.060). (D) Patients with high CD11c expression showed a better OS in cervical cancer (Log‐rank test, *p* = 0.021). (E) Patients with high CD24/CD47 and low CD11c expression exhibited a worse OS (Log‐rank test, *p* = 0.008, *p* < 0.001, respectively).

The ImmuCellAI database was used to explore the tumor microenvironment in cervical cancer. A variety of tumor‐infiltrating immune cells in the cervical cancer microenvironment were calculated (Figure 2A), among which macrophages, NK cells, T central memory (Tcm) cells, and T follicular helper (Tfh) cells were predominant. Previous studies have illustrated that the abundance of infiltrated macrophages is associated with clinical outcomes in cervical cancer. The TCGA cohort was dichotomized by the infiltration level of total macrophages using the optimal cutoff point. However, the survival advantage in the high macrophage infiltration group was not statistically significant (*p* = 0.106, Figure 2B). To further investigate the negative result, we evaluated the prognostic value of M1‐polarized and M2‐polarized macrophages. Patients with a high M1/M2 ratio had a better prognosis although the difference did not reach statistical significance (*p* = 0.060, Figure 2C). Considering that M1/M2‐polarized macrophages are not routinely counted in clinical pathological tests, we searched for simpler markers, CD11c and CD206, which roughly estimate the abundance of infiltrated M1‐ and M2‐polarized macrophages, respectively. Interestingly, CD11c was a prognosticator for better survival in cervical cancer (*p* = 0.021, Figure 2D) and exhibited a superior discriminative ability. However, CD206 didn't serve as an indicator of prognosis (*p* = 0.200, Figure S2B).

Furthermore, we incorporated CD11c into survival analysis with CD24 and CD47 expression, respectively. When combined with CD11c expression level, CD24/CD47 showed a better ability to predict clinical outcomes. Tumors with high CD24/CD47 expression and simultaneous low CD11c expression level demonstrated the worst OS. In contrast, ones with low CD24/CD47 expression and high CD11c expression level predicted the best (*p* = 0.008, *p* < 0.001, respectively, Figure 2E). As for CD47, overexpression alone showed a trend toward worse (Figure 1D), but statistical significance was not reached. In order to elucidate the role of CD11c in the prognostic value of CD47, a subgroup analysis was conducted. The findings indicated that in the CD47 low expression group, CD11c expression levels exhibited no correlation with OS, while in the CD47 high expression group, low CD11c expression levels demonstrated a positive correlation with enhanced OS (*p* < 0.001, Figure S2C). Furthermore, it was observed that the clinical significance of CD47 only emerges in conjunction with CD11c status.

### 3.3 CD24 and CD47 Expression in Cervical Cancers From FUSCC

**FIGURE 3:**
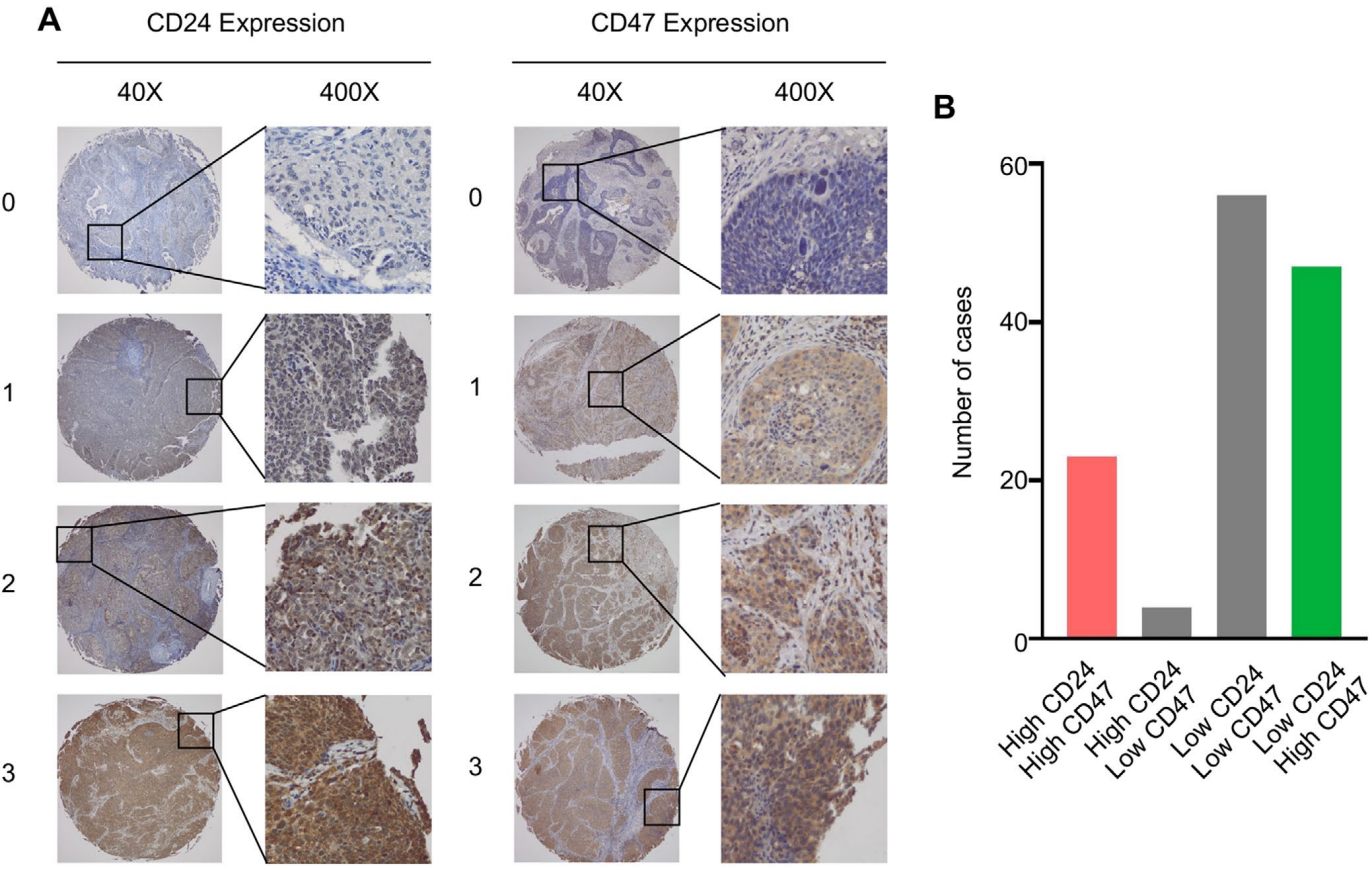
CD24 and CD47 in FUSCC CC by IHC on a TMA. (A) Representative images of CD24 (left) and CD47 (right) expression in cervical cancers (stained by IHC). (B) Distribution of cervical cancers with different expression levels of CD24 and CD47 (*n* = 130). Cutoff values: High CD24 expression is defined as an H‐score ≥ 180; high CD47 expression is defined as an H‐score ≥ 90, with the optimal cutoff point determined by the R package “surv_cutpoint”.

**FIGURE 4:**
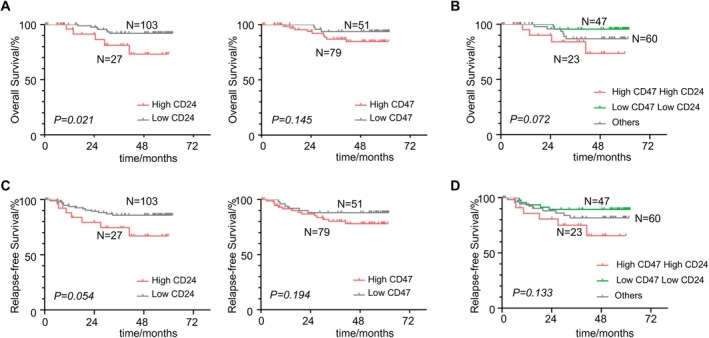
CD24 and CD47 expression in FUSCC CC and corresponding association with OS and RFS. (A) High expression of CD24 was associated with poor OS (Log‐rank test, *p* = 0.021). The correlation between OS and the expression level of CD47 wasn't significant (Log‐rank test, *p* = 0.145) in the Kaplan–Meier analysis of CC from FUSCC (*n* = 130). (B) High expression of both CD24 and CD47 didn't show a significant association with a poor OS (Log‐rank test, *p* = 0.072). (C) Patients with high expression levels of CD24/CD47 showed a tendency of poor RFS (Log‐rank test, *p* = 0.058, *p* = 0.194, respectively). (D) High expression of both CD24 and CD47 didn't show a significant association with a poor RFS (Log‐rank test, *p* = 0.133).

We further investigated a clinically annotated CC tissue microarray by immunohistochemistry staining against CD24/CD47 to validate their capacity as a prognostic biomarker. Representative images of IHC staining against CD24/CD47 are shown in Figure 3A. According to the optimal cutoff values of protein expression level (180 for CD24; 90 for CD47), 130 CC cases were assigned to four groups, the distribution of which is displayed in Figure 3B. Clinicopathological characteristics and associations between CD24/CD47 expression are presented in Table 1. Among the 130 patients, 45 were classified in FIGO stage I, and 85 were in FIGO stage II. All 130 cases had undergone radical hysterectomy and pelvic lymphadenectomy. After the operation, 40 patients received chemotherapy and 51 patients received postoperative radiation therapy because of positive pelvic lymph nodes, parametrial invasion, or positive surgical margins. Consistent with the results of TCGA, we detected that high CD24 expression level exhibited superior predictive capacity for worse OS (*p* = 0.021, Figure 4A) and demonstrated a strong trend toward worse RFS (*p* = 0.054, Figure 4C). High CD47 expression showed a trend toward worse prognosis in both OS (*p* = 0.145, Figure 4A) and RFS (*p* = 0.194, Figure 4C), though these differences did not reach statistical significance. We then integrated both CD24 and CD47 into the analysis. Unfortunately, further stratification did not show any superiority to CD24 alone regarding prognosis prediction (*p* = 0.072, Figure 4B; *p* = 0.133, Figure 4D, respectively), although patients with high expression of both CD24 and CD47 were prone to undesirable outcomes. A univariate analysis indicated that tumor histology (*p* = 0.041) and CD24 expression level (*p* = 0.021) were risk factors for OS. A multivariate Cox proportional model identified CD24 expression level as an independent prognostic factor. Compared with the low expression group, patients in the high expression group were more likely to have worse OS (HR 4.179, 95% CI 1.095–15.948, *p* = 0.036, Table 2).

**TABLE 1:** Demographic and histopathologic characteristics of cervical cancer patients with different CD24 or CD47 expression levels.

Variables	Total	CD24 expression		*p*	CD47 expression		*p*
		High	Low		High	Low	
Age (mean ± SD)	49.5 ± 9.0	27	103		79	51	
FIGO stage				0.120			0.188
I	46	6	40		24	22	
II	84	21	63		55	29	
Tumor size (cm)				0.666			0.718
≤ 4	73	14	59		43	30	
> 4	57	13	44		36	21	
Histology				0.039*			0.413
SCC	114	20	94		69	45	
AC	10	5	5		5	5	
Others	6	2	4		5	1	
Depth				0.998			0.609
≤ 1/3	15	3	12		8	7	
> 1/3 and ≤ 2/3	44	9	35		25	19	
> 2/3	70	14	56		45	25	
NA	1	1	0		1	0	
LN metastasis				0.244			0.244
No	90	16	74		58	32	
Yes	40	11	29		21	19	
LVSI				0.084			0.856
No	71	11	60		43	28	
Yes	56	16	40		35	21	
NA	3	0	3		1	2	

Abbreviations: AC, adenocarcinoma; LN metastasis, lymph‐node metastasis; LVSI, lymphovascular space invasion; SCC, squamous cell carcinoma.

* *p* < 0.05.

**TABLE 2:** Univariate and multivariate analysis of determinants of overall survival in cervical cancer patients.

Variables	Univariate analysis			Multivariate analysis		
	3‐year CSS (%)	Log‐rank *χ* ^2^	*p*	HR	95% CI	*p*
Age (years)		0.789	0.374			0.816
< 50	91.60			1.000	Reference	
≥ 50	88.00			0.837	0.188–3.731	
FIGO stage		2.769	0.096			0.574
I	95.20			1.000	Reference	
II	86.70			1.647	0.289–9.378	
Tumor size (cm)		1.611	0.204			0.363
≤ 4	92.00			1.000	Reference	
> 4	87.30			1.955	0.462–8.272	
Histology		6.388	0.041*			0.011*
SCC	90.60			1.000	Reference	
AC	100.00			0.547	0.060–4.948	
Others	60.00			24.722	2.924–209.019	
Depth		5.205	0.074			0.085
≤ 1/3	91.70			1.000	Reference	
> 1/3 and ≤ 2/3	97.30			0.038	0.001–1.266	
> 2/3	84.60			0.652	0.056–7.602	
LN metastasis		2.368	0.124			0.587
No	93.50			1.000	Reference	
Yes	82.00			1.570	0.309–7.986	
LVSI		1.256	0.262			0.199
No	93.30			1.000	Reference	
Yes	84.80			3.162	0.545–18.348	
CD47		2.122	0.145			0.152
Low	93.80			1.000	Reference	
High	86.90			2.901	0.676–12.443	
CD24		5.317	0.021*			0.036*
Low	92.10			1.000	Reference	
High	81.30			4.179	1.095–15.948	

Abbreviation: CSS, cancer‐specific survival.

* *p* < 0.05.

## 4 Discussion

Immune checkpoint blockade has brought hope for the treatment of patients with recurrent or advanced cervical cancer. The majority of current studies have mainly focused on adaptive immune checkpoints, such as PD‐1/PD‐L1 and CTLA‐4/B7. However, it is reported that the most abundant leukocyte population is tumor‐associated macrophages (TAMs) in solid tumors [23], thus targeting TAMs may offer better immunotherapeutic treatment for solid tumors. Macrophage‐mediated phagocytosis is mainly controlled by prophagocytic and antiphagocytic signals on the surface of target cells [24]. The overexpression of antiphagocytic signals (“don't eat me”) has been correlated with tumor progression, such as signaling involving CD47 and CD24.

The current study was performed to examine the therapeutic potential of CD24 and CD47 blockade in cervical cancer. We found that both CD24 and CD47 were overexpressed in cervical cancers, and high CD24 expression predicted a worse prognosis in both TCGA and FUSCC cohorts while CD47 expression did not show significant predictive ability in either cohort. CD24 was originally identified as involved in a series of inflammatory responses through binding with Siglec‐10 on innate immune cells, including in infection, sepsis, and liver damage [25, 26, 27]. Its function in regulating tumor immunity was recently discovered by Amira et al. [11], who found that CD24 blockade suppressed tumor growth in ovarian and breast cancer. CD47 acts as an innate immune checkpoint to regulate homeostatic phagocytosis. The absence of CD47 in senescent or damaged cells can trigger macrophage phagocytosis. Upregulation of CD47 in cancer cells can allow them to evade phagocytosis, and this process is harnessed by cancer cells to induce immune suppression. CD47 is overexpressed in various cancer cells and correlates with distant metastases. Brightwell et al. reported that CD47 is expressed at high frequency in human ovarian cancer, and patients with low expression of CD47 tend to have a better treatment response to standard therapy [28]. Similar findings have been demonstrated in other human cancers, including gastric cancer, liver cancer, lung cancer, acute myeloid leukemia, non‐Hodgkin lymphoma (NHL), and breast cancer [29, 30, 31, 32, 33, 34]. The authors showed that blocking CD24 and CD47 enhanced phagocytosis additively and was better than CD47 blockade alone. Anti‐CD24 treatment can still be effective in cancers that have been resistant to CD47 blockade. Furthermore, our work demonstrated CD24 expression as an independent prognostic predictor in patients with cervical cancer, while the discriminative ability of CD47 expression did not reach statistical significance, which may indicate that the CD24‐Siglec‐10 signal plays a more vital role than the CD47‐SIRPα signal to help tumor cells evade immune surveillance in cervical cancer.

Macrophages play an essential role in modulating the tumor immune microenvironment, as they eliminate both exogenous and endogenous pathogens to promote adaptive immune response [35]. Existing studies believe that classically activated M1 macrophages are powerful killers of cancer cells, and M2 macrophages, referred to as TAMs, usually inhibit anti‐tumor responses executed by cytotoxic lymphocytes [36]. Upon receiving the “don't eat me” signal, macrophages undergo M2 polarization and exhibit an immune inhibitory phenotype, which reduces the anti‐tumor response of cytotoxic lymphocytes. It is reported that as the tumor progressed, the abundance of TAMs increased, while that of M1 macrophages decreased [37]. More and more evidence shows that a large portion of cervical cancers, reaching up to 78%, manifest with a high abundance of immune cell infiltration, which is dominated by macrophages and T cells [38]. According to the above discussion, we may speculate that it is too cursory to evaluate innate immune status based on expression levels of immune‐related molecules, such as CD24 and CD47. The innate immune status of cervical cancer is a multifaceted phenomenon that is subject to regulation by a variety of factors, including the abundance of M1 macrophages, the proportion of macrophages, the M2/M1 ratio, and other immune checkpoint. In this research, we found that a high M1/M2 ratio could be a potential prognostic factor for improved survival in cervical cancer. Additionally, patients with high expression of CD11c showed a better prognosis, a well‐known biomarker of M1‐polarized macrophages. Thus, a high abundance of M1‐polarized macrophages can be considered a sign of a better prognosis in cervical cancer, and CD11c is a potential prognostic factor that can be employed as a simpler alternative to M1‐polarized macrophages.

Therefore, we further incorporated the abundance of macrophages and CD11c into survival analysis, together with the expression level of CD24 and CD47. Interestingly, patients with high expression of CD24/CD47 and low infiltration levels of M1‐polarized macrophages show the worst survival. The key to establishing an effective anti‐cancer therapy is to identify the specific patient group that can benefit the most from the treatment. From this perspective, combining M1‐polarized macrophage infiltration and CD24/CD47 expression is more effective than CD24/CD47 alone in classifying cervical cancer patients. In contrast to CD24, which exhibits prognostic implications for cervical cancer irrespective of its association with CD11c, the prognostic value of CD47 is contingent on CD11c, particularly the abundance of infiltrating macrophages within the cervical cancer microenvironment. This discrepancy may be attributable to the observation that CD47‐mediated immune evasion is contingent on its interaction with SIRPA on tumor‐associated macrophages [39, 40]. Concurrently, CD24 facilitates the immune evasion of tumor cells by recognizing Siglec‐G/10 on macrophages, P‐/E‐selectin on endothelial cells, and L1CAM on lymphocytes [41].

Tumor cells can evade TAM clearance by overexpressing antiphagocytic surface proteins known as “don't eat me” signals, such as, CD47 and CD24. Blocking these “don't eat me” signals with antibodies has demonstrated therapeutic potential across various cancers. CD47 is a prominent target of the “don't eat me” pathway. Numerous CD47‐specific inhibitors, including magrolimab (Hu5F9‐G4), evorpacept, AK117, and IMM01, have been developed and are undergoing clinical trials for hematologic and solid tumors [42, 43]. However, as CD47 monoclonal antibodies such as magrolimab have demonstrated an absence of efficacy in pivotal clinical trials for hematologic malignancies and have exhibited significant hematologic toxicity, clinical research targeting CD47 faces challenges [44, 45]. The optimization of the structure of CD47 antibodies to reduce their affinity for erythrocytes and the exploration of combination therapies are two emerging new avenues for CD47‐targeted therapies. Several studies have confirmed that the blockade of the CD47–SIRPα pathway is capable of enhancing the anti‐PD‐L1 therapy effect in melanoma [46, 47]. Unlike CD47, CD24 has a more restricted distribution in healthy tissues and higher expression in tumor tissues. Furthermore, it has not been expressed on human erythrocytes [48]. This reduces on‐target and off‐tumor toxicity (OTOT), resulting in a wider safety window for CD24‐targeted therapies. Multiple preclinical studies have demonstrated that CD24 monoclonal antibodies can effectively inhibit the proliferation of cancer cells, including breast and ovarian cancers [49, 50]. Currently, several CD24 monoclonal antibodies, including ATG‐031 (NCT04986865, NCT06028373), IMM47 (NCT05985053), PHST001 (NCT06840886), and KH801 (NCT06364501), have been approved for Phase I clinical trials in solid tumors. In addition, CD24‐targeted antibody‐drug conjugates (ADCs), chimeric antigen receptor T cell (CAR‐T) therapies, and nanomedicines are under investigation. Interestingly, preclinical studies of ATG‐031 have shown that ATG‐031‐induced phagocytosis transforms macrophages from a tumor‐tolerant M2 phenotype to an antitumor M1 phenotype. This finding provides a theoretical basis for the combined therapy of CD24 and CD47 in solid tumors.

This study is the first report investigating dual CD47 and CD24 expressions and comparing their correlation with clinicopathologic and prognostic characteristics in cervical cancer. Our study had several limitations. First, CD47 and CD24 expressions were simply detected with IHC in the FUSCC cohort, while expression data from TCGA were based on RNA sequencing. Further studies should examine the expressions of CD47 and CD24 at the transcriptional levels to further understand the mechanism of CD47/SIRPα and CD24/Siglec‐1 pathways in the anti‐tumor immune response. Second, although this study mainly explored the clinical translational value of blocking CD24 and CD47 signals to increase the anti‐tumor immune response of macrophages, this study also found that, in addition to macrophages, the cervical cancer microenvironment was rich in infiltration of dendritic cells, NK cells, and CD8+ T cells (Figure 2A). However, this study lacked further exploration of the role and clinical translational value of other immune cells and checkpoints in cervical cancer. There is hence a pressing need for identifying and quantifying the immune landscape of cervical cancer in our further research. Thirdly, although the TCGA and FUSCC cohorts were combined to enhance statistical power, the TCGA dataset has significant limitations regarding the completeness of clinical variables. A salient issue is the paucity of surgical pathology parameters (including tumor size, depth of invasion, lymph node metastasis status, and the presence of lymph vascular space invasion) in the majority of cases, as only a subset of patients underwent curative surgery. These omissions, however, imposed limitations on our capacity to undertake multivariate analyses and adjust for established prognostic factors. Finally, the impact of this study is somewhat limited by the retrospective design and relatively small sample size; future prospective studies with more patients are required to elucidate the relationships between the CD24/CD47 expression levels, macrophage abundance, and their prognostic influence.

## 5 Conclusions

The expression level of CD24 is an independent prognostic factor for cervical cancer. The high CD47 expression and low M1‐polarized macrophage infiltration predict a worse prognosis. The study provides potential prognostic markers and increases the feasibility of CD24 and CD47 blockade in cervical cancer treatment.

## Author Contributions

**Min Yu:** investigation, writing – original draft, visualization, software, conceptualization. **Mengdong Ni:** funding acquisition, writing – original draft, methodology, software. **Fei Xu:** investigation, visualization, writing – original draft, validation. **Chi Fang:** formal analysis, data curation. **Jiajia Li:** formal analysis, data curation, project administration. **Xiaohua Wu:** supervision, writing – review and editing, project administration, resources. **Guihao Ke:** writing – review and editing, funding acquisition, supervision, resources.

## Ethics Statement

The clinical samples and information used in the study were approved by the Research Ethics Committee of Fudan University Shanghai Cancer Center (FUSCC).

## Conflicts of Interest

The authors declare no conflicts of interest.

## Supporting information


**Figure S1:** The correlation of CD24 and CD47 expression levels. (A) CD24 and CD47 expression didn't show significant differences among different tumor stages in cervical cancer. (B) CD47 expression was positively related to CD24 expression in cervical cancer by GEPIA database analysis (purity‐adjusted Spearman's rho = 0.19, *p* < 0.001).
**Figure S2:** Prognostic value of macrophage infiltration level and its correlation with CD24/CD47 expression. (A) Patients with high expression level of CD24 and low infiltration level of macrophages showed a poor but insignificant OS (Log‐rank test, *p* = 0.060). Patients with high expression level of CD47 and low infiltration level of macrophages exhibited a worse OS (Log‐rank test, *p* = 0.020). (B) Patients with different CD206 expression levels had no significant difference in OS (Log‐rank test, *p* = 0.200). (C) Patients with high CD11c expression had a better OS in the CD47‐high subgroup (Log‐rank test, *p* < 0.001). Kaplan–Meier curves according to CD11c expression (M1) in the CD47‐low and CD47‐high subgroups.

## Data Availability

The data that support the findings of this study are available from the corresponding author upon reasonable request.
