# Supplementary figures and images for: Overexpressed CD24 and CD47 Indicate a Worse Prognosis in Cervical Cancer
Source: Cancer Med. 2025 Dec 7;14(23):e71443. doi: 10.1002/cam4.71443 (PMC12682362; doi:10.1002/cam4.71443)

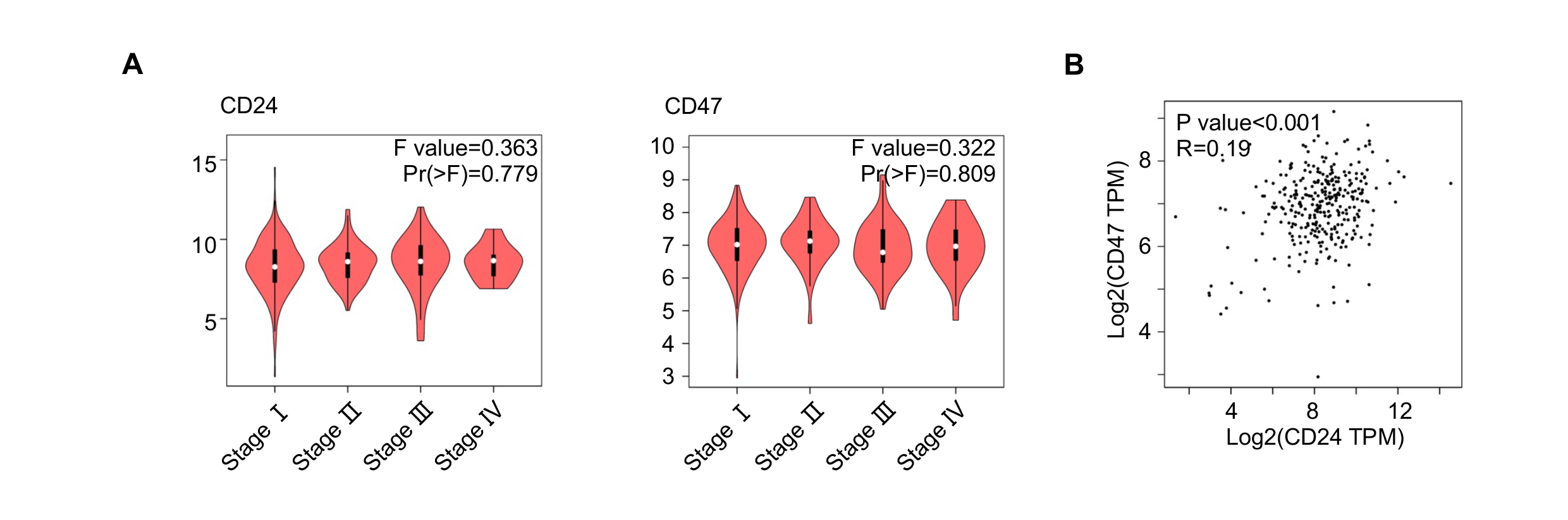

Supplement: Supplementary file 1 — Figure S1: The correlation of CD24 and CD47 expression levels. (A) CD24 and CD47 expression didn't show significant differences among different tumor stages in cervical cancer. (B) CD47 expression was positively related to CD24 expression in cervical cancer by GEPIA database analysis (purity‐adjusted Spearman's rho = 0.19, p < 0.001). Figure S2: Prognostic value of macrophage infiltration level and its correlation with CD24/CD47 expression. (A) Patients with high expression level of CD24 and low infiltration level of macrophages showed a poor but insignificant OS (Log‐rank test, p = 0.060). Patients with high expression level of CD47 and low infiltration level of macrophages exhibited a worse OS (Log‐rank test, p = 0.020). (B) Patients with different CD206 expression levels had no significant difference in OS (Log‐rank test, p = 0.200). (C) Patients with high CD11c expression had a better OS in the CD47‐high subgroup (Log‐rank test, p < 0.001). Kaplan–Meier curves according to CD11c expression (M1) in the CD47‐low and CD47‐high subgroups. [file CAM4-14-e71443-s001.zip › cam471443-sup-0001-FigureS1@Figure S1.jpg]

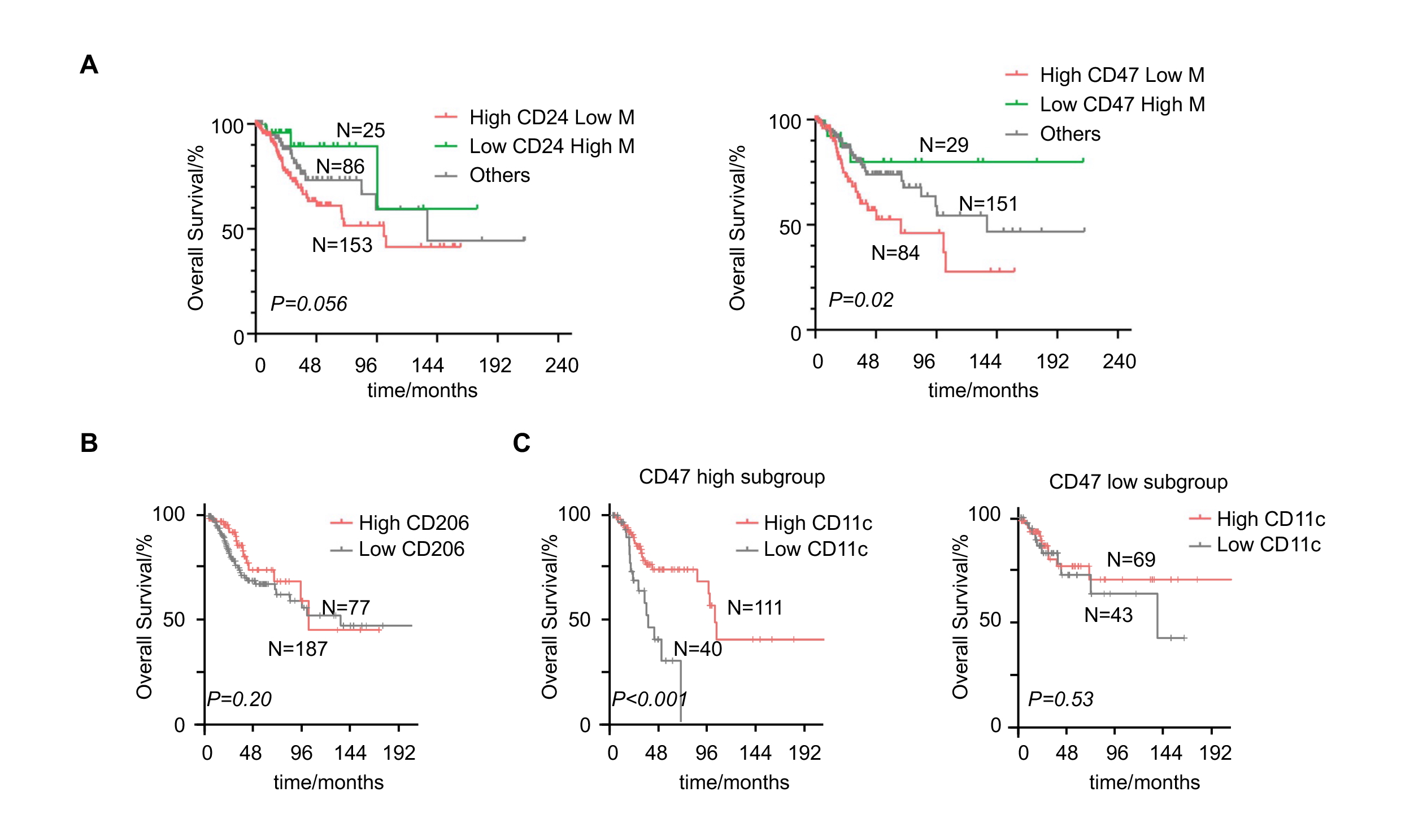

Supplement: Supplementary file 1 — Figure S1: The correlation of CD24 and CD47 expression levels. (A) CD24 and CD47 expression didn't show significant differences among different tumor stages in cervical cancer. (B) CD47 expression was positively related to CD24 expression in cervical cancer by GEPIA database analysis (purity‐adjusted Spearman's rho = 0.19, p < 0.001). Figure S2: Prognostic value of macrophage infiltration level and its correlation with CD24/CD47 expression. (A) Patients with high expression level of CD24 and low infiltration level of macrophages showed a poor but insignificant OS (Log‐rank test, p = 0.060). Patients with high expression level of CD47 and low infiltration level of macrophages exhibited a worse OS (Log‐rank test, p = 0.020). (B) Patients with different CD206 expression levels had no significant difference in OS (Log‐rank test, p = 0.200). (C) Patients with high CD11c expression had a better OS in the CD47‐high subgroup (Log‐rank test, p < 0.001). Kaplan–Meier curves according to CD11c expression (M1) in the CD47‐low and CD47‐high subgroups. [file CAM4-14-e71443-s001.zip › cam471443-sup-0003-FigureS2@Figure S2.jpg]
